# Esophageal Bypass Surgery for an Esophagobronchial Fistula Following Palliative Irradiation for Left Main Bronchial Obstruction Caused by Mediastinal Malignant Lymphoma

**DOI:** 10.70352/scrj.cr.25-0246

**Published:** 2025-09-17

**Authors:** Byonggu An, Tetsuya Abe, Yasumitsu Oe, Yuki Morimoto, Yuuki Imazato, Hibiki Kanda, Katsunori Tai, Hiroki Fujita, Toru Imagami, Takeshi Togawa, Nobuyuki Takao, Akira Sogawa, Takashi Matsunaga, Shizuki Takemura, Akiyoshi Mizumoto

**Affiliations:** 1 Department of Digestive Surgery, Omi Medical Center, Kusatsu, Shiga, Japan; 2 Department of Gastroenterological Surgery, Aichi Cancer Center Central Hospital, Nagoya, Aichi, Japan; 3 Department of Respiratory Medicine, Omi Medical Center, Kusatsu, Shiga, Japan; 4 Department of Hematology, Omi Medical Center, Kusatsu, Shiga, Japan; 5 Department of Gastroenterology, Omi Medical Center, Kusatsu, Shiga, Japan; 6 Department of Diagnostic Pathology, Omi Medical Center, Kusatsu, Shiga, Japan

**Keywords:** central airway obstruction (CAO), esophagobronchial fistula (EBF), palliative radiotherapy, esophageal bypass surgery

## Abstract

**INTRODUCTION:**

Central airway obstruction (CAO) due to malignancy is an oncological emergency that requires prompt management. On the other hand, esophagobronchial fistula (EBF) is also a serious condition that can lead to aspiration pneumonia and difficulty with oral intake. Secondary involvement due to malignant lymphoma is a very rare cause of both CAO and EBF. Here, we report a case of esophageal bypass surgery for an EBF that became apparent after palliative irradiation for left main bronchial obstruction caused by mediastinal malignant lymphoma.

**CASE PRESENTATION:**

A 76-year-old man was referred to our hospital with a chief complaint of dyspnea and dysphagia. Contrast-enhanced chest CT revealed a 10-cm irregularly shaped space-occupying lesion (SOL) in the middle mediastinum, involving the left main bronchus and esophagus. The left main bronchus was obstructed by the SOL, and the presence of gas within the lesion suggested the formation of a fistula between the tumor and the bronchus. Bronchoscopy revealed almost complete obstruction of the left main bronchus due to an ulcerative tumor. Upper gastrointestinal endoscopy revealed a deeply excavated esophageal ulcer, raising suspicion of a fistulous connection with the tumor. Although fistula formation between the tumor and the bronchus and/or esophagus was suspected, due to the oncological emergency of CAO presumably caused by mediastinal-type lung cancer, palliative irradiation and intravenous administration of betamethasone sodium phosphate were initiated prior to obtaining a pathological diagnosis. The tumor shrank remarkably after the treatment. However, as the tumor regressed, an EBF became apparent. Given the patient’s strong desire to resume oral intake and the need for a definitive diagnosis, we planned a surgical intervention (esophageal bypass surgery with lymph node sampling). The patient’s postoperative course was uneventful. He resumed oral intake on POD 9, and was discharged on day 20. Histopathological examination of the lymph nodes revealed diffuse large B-cell lymphoma. The patient began chemotherapy for malignant lymphoma 2 months after surgery.

**CONCLUSIONS:**

Esophageal bypass surgery is a valuable option for patients with an EBF, as it improves QOL and enables subsequent chemotherapy.

## Abbreviations

CAO
: central airway obstruction
CD20
: cluster of differentiation 20
CRP
: C-reactive protein
CRT
: chemoradiotherapy
EBF
: esophagobronchial fistula
ECOG-PS
: Eastern Cooperative Oncology Group performance status
ERFs
: esophagorespiratory fistulas
FDG-PET/CT
: ^18^F-fluorodeoxyglucose PET/CT
N/C
: nuclear-to-cytoplasmic
SOL
: space occupying lesion

## INTRODUCTION

Oncological emergencies are defined as acute conditions caused by a cancer or cancer treatment that require prompt intervention to prevent death or serious complications.^1)^ Among oncological emergencies, CAO due to a malignancy is a critical condition that requires early intervention.^1,2)^ Treatment options for CAOs due to malignant tumors include steroid therapy and emergency irradiation to maintain the airway. However, tumor shrinkage may lead to the development of a fistula.^1,3)^ In particular, an EBF can severely impair oral intake, which leads to a major deterioration in the patient’s QOL, and places patients at risk of pneumonia due to aspiration of saliva.^4)^ Here, we report a patient who underwent esophageal bypass surgery for an EBF that revealed after palliative irradiation for left main bronchus obstruction due to mediastinal malignant lymphoma.

## CASE PRESENTATION

**Fig. 1:**
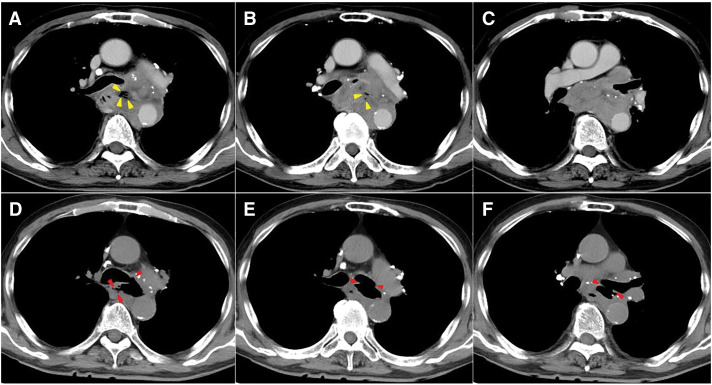
(**A**–**C**) CT showed a 10 cm-sized, irregularly shaped space-occupying lesion (SOL) involving the left main bronchus and esophagus. The left main bronchus was obstructed by the SOL, and gas was present within the lesion (yellow arrowheads). (**D**–**F**) The tumor shrank remarkably after radiation treatment. However, the esophagobronchial fistula became apparent (red arrowheads).

**Fig. 2:**
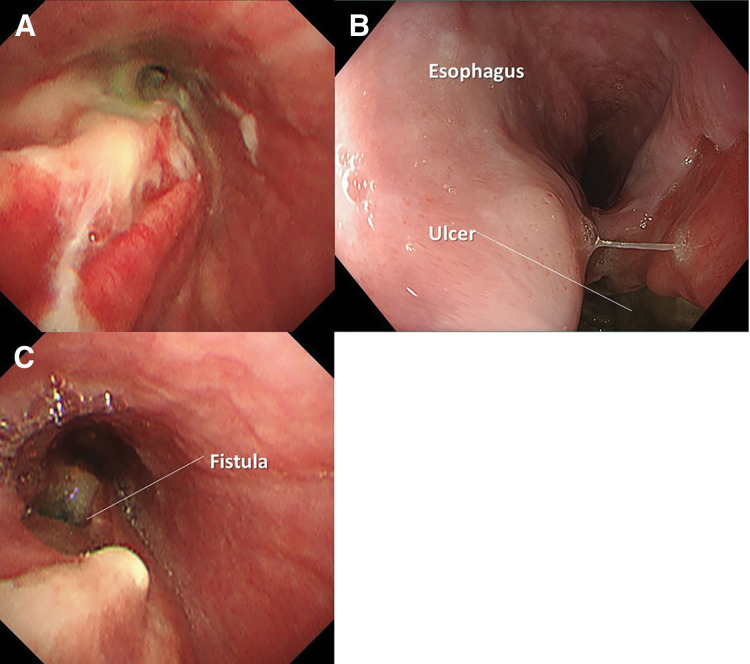
(**A**) Bronchoscopy revealed stenosis of the left main bronchus due to an ulcerative tumor. (**B**) Upper gastrointestinal endoscopy revealed a deeply excavated esophageal ulcer, raising suspicion of a fistulous connection with the tumor. (**C**) Significant shrinkage of the tumor following palliative irradiation revealed an esophagobronchial fistula.

**Fig. 3:**
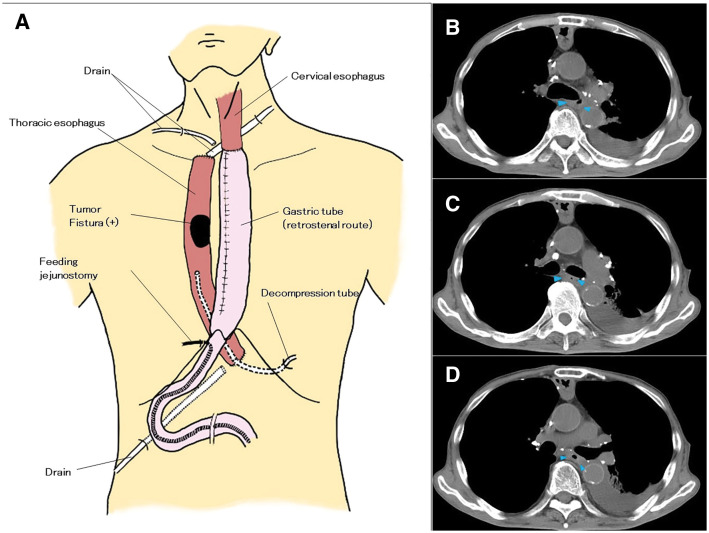
(**A**) Schematic diagram of the esophageal bypass surgery. (**B**–**D**) Follow-up CT 4 months postoperatively was negative for complications such as an abscess or mucocele in the thoracic esophagus (blue arrowheads).

A 76-year-old man was referred to our hospital with a chief complaint of dysphagia and dyspnea. He had a history of tuberculosis in childhood. Contrast-enhanced chest CT revealed a 10-cm irregularly shaped SOL in the middle mediastinum, involving the left main bronchus and esophagus (**Fig. 1A**–**1C**). The left main bronchus was obstructed by the SOL, and the presence of gas within the lesion suggested the formation of a fistula between the tumor and the bronchus. On admission, the patient’s vital signs were stable. Laboratory data suggested an elevated inflammatory response (white blood cells: 9800/μL, CRP: 3.28 mg/dL) and abnormally high lactate dehydrogenase levels (614 U/L). Bronchoscopy revealed almost complete obstruction of the left main bronchus due to an ulcerative tumor (**Fig. 2A**). Upper gastrointestinal endoscopy revealed a deeply excavated esophageal ulcer, raising suspicion of a fistulous connection with the tumor (**Fig. 2B**). Although fistula formation between the tumor and the bronchus and/or esophagus was suspected, due to the oncological emergency of CAO presumably caused by mediastinal-type lung cancer, palliative irradiation (30 Gy in 10 fractions) and intravenous administration of betamethasone sodium phosphate (8 mg/day) were initiated prior to obtaining a pathological diagnosis. The tumor shrank remarkably after the treatment. However, as the tumor regressed, an EBF became apparent (**Figs. 1D**–**1F** and **2C**), necessitating the cessation of oral intake. The patient was managed with antibiotics and central venous nutrition. Results of the bronchoscopic biopsy suggested malignant lymphoma, but a definitive diagnosis could not be established because of the small specimen. Subsequent FDG-PET/CT revealed newly enlarged lymph nodes along the lesser curvature of the stomach, raising suspicion of lymph node metastases (**Supplementary Fig. A–C**). Given the patient’s strong desire to resume oral intake and the need for a definitive diagnosis, we planned a surgical intervention (esophageal bypass surgery with lymph nodes sampling) (**Fig. 3A**).

The patient was placed in the supine position under general anesthesia. His abdomen was opened, and the abdominal esophagus was divided above the esophagogastric junction, with the esophageal stump closed by linear staplers (Signia stapling system; Medtronic Japan, Tokyo, Japan). During the creation of a gastric tube, the lesser curvature of the stomach was resected along with lymph nodes that had been identified on preoperative FDG PET-CT. The cervical esophagus was then divided above the sternal notch, and the esophageal stump was closed by linear staplers. The gastric tube was pulled up through the retrosternal route, and a hand-sewn technique was used to anastomose the gastric tube to the cervical esophagus. An abdominal approach was used to place a decompression tube in the remnant esophagus through the distal esophageal stump, and a feeding jejunostomy tube was added for nutritional management. The patient’s postoperative course was uneventful. He resumed oral intake on POD 9, and was discharged on day 20. The drainage from the decompression tube was minimal, and was removed 6 weeks after surgery. Follow-up CT 4 months postoperatively was negative for complications such as an abscess or mucocele in the thoracic esophagus (**Fig. 3B**–**3D**).

**Fig. 4:**
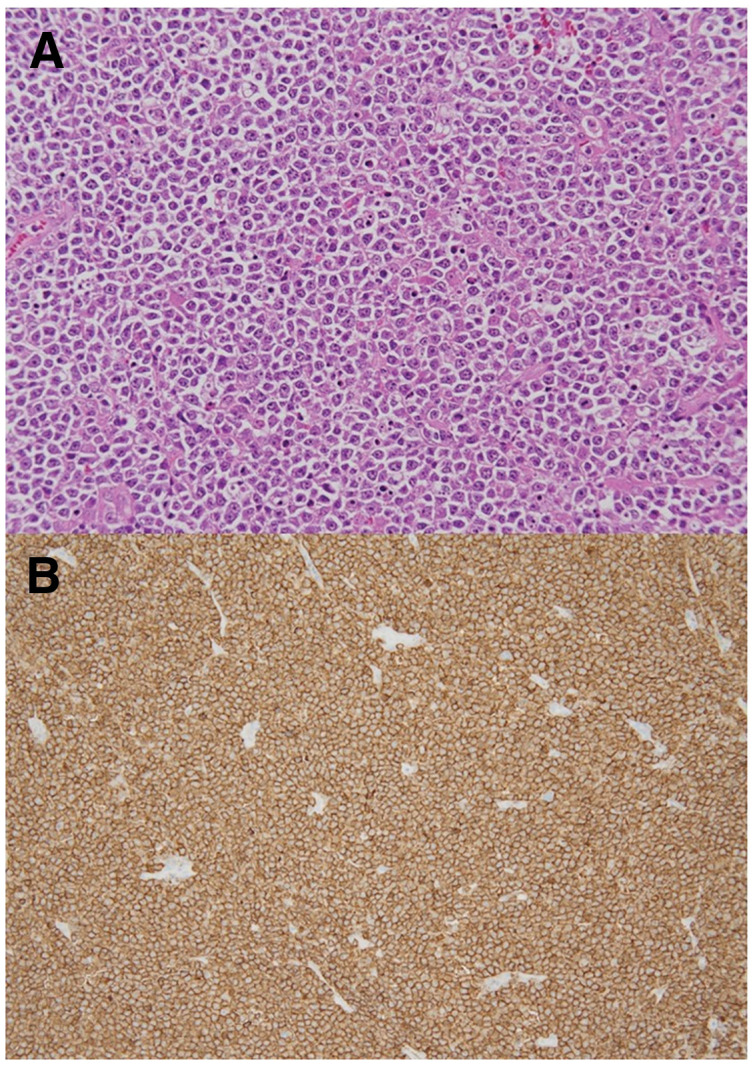
(**A**) Histopathological examination of the lymph nodes revealed diffusely proliferating large cells with round nuclei and a high nuclear-to-cytoplasmic (N/C) ratio. (**B**) Immunohistochemical analysis was positive for cluster of differentiation 20 (CD20) expression.

Histopathological examination of the lymph nodes revealed diffusely proliferating large cells with round nuclei and a high N/C ratio (**Fig. 4A**). Immunohistochemical analysis was positive for CD20 expression, leading to a final diagnosis of diffuse large B-cell lymphoma (**Fig. 4B**). The patient began chemotherapy for malignant lymphoma 2 months postoperatively and remains alive at the time of this writing 8 months after surgery.

## DISCUSSION

Our case report highlights 2 major clinical points. First, palliative radiotherapy for mediastinal malignant lymphoma with stenosis of the left main bronchus helped prevent an oncological emergency. Second, we successfully performed esophageal bypass surgery for an EBF caused by mediastinal malignant lymphoma, enabling the patient to resume oral intake and receive postoperative chemotherapy.

CAO is defined as an obstruction of the major airways, namely, the trachea and main bronchi.^1,2)^ CAO can result from various benign and malignant diseases, among which airway narrowing due to malignancy is an oncological emergency requiring prompt management. Lung cancer is the most common cause of CAO resulting from malignant tumors, whereas malignant lymphoma is relatively rare.^2,3,5)^ In our patient, contrast-enhanced CT and bronchoscopy revealed a tumor-related obstruction of the left main bronchus, which was immediately thought to be CAO due to lung cancer. Therefore, to avoid an oncological emergency, we performed palliative irradiation without waiting for the pathological diagnosis. Palliative-intent radiation administered concomitant with steroid therapy is effective for opening obstructed airways.^1)^ In addition, malignant lymphomas are highly radiosensitive and require less radiation than conventional solid tumors to achieve local control.^6)^ Since the histopathological diagnosis was diffuse large B-cell lymphoma, the tumor shrank remarkably after radiation treatment, and an oncological emergency was prevented.

However, as the tumor regressed, an EBF became apparent. EBFs associated with lymphomas typically develop during or after radiotherapy or chemotherapy, and rarely develop before treatment.^3)^ In our patient, we initially suspected the presence of a bronchial or esophageal fistula because the tumor appeared to be invasive, as the CT scan showed gas within the tumor. Additionally, the bronchoscopic and upper gastrointestinal endoscopic findings suggested the presence of a fistula. However, since the left bronchus was obstructed by the tumor, palliative irradiation was prioritized to alleviate the patient’s signs of respiratory distress, and subsequently led to the more apparent development of an EBF. In oncological emergencies, particularly CAO, palliation of respiratory distress should be the primary consideration when selecting a treatment.

An EBF is a serious condition. The QOL of patients with EBF is severely impaired, and they often manifest dysphagia and dyspnea and develop aspiration pneumonia and sepsis, all of which require immediate interventions.^4)^ Management of EBFs caused by malignancy is primarily palliative. Extensive surgical repair is usually not performed, and a minimally invasive approach is preferred.^7)^ Stenting or bypass surgery is often the treatment of choice for an EBF. There have been many recently published reports on stents being used because the stent procedure is minimally invasive and easy to perform.^7,8)^ However, stent placement is generally not indicated in the following patients: (1) those with tumors located in the cervical esophagus or upper thoracic region; (2) those with complete tumor-induced obstruction that prevents passage of a guidewire; (3) those with large fistulas; (4) those with short or only mildly stenotic lesions, due to the risk of stent migration; and (5) those scheduled to undergo CRT in the near future.^8,9)^ Notably, stent placement performed either before or after radiotherapy has been associated with a heightened risk of severe complications, including enlargement of the fistula, esophageal rupture, and massive hematemesis.^10)^ The 2021 clinical guidelines issued by the European Society of Gastrointestinal Endoscopy do not recommend the combined use of palliative external beam radiotherapy and esophageal stenting.^11)^ Furthermore, several reports have indicated that combining stent insertion with CRT increases the incidence of such adverse events.^8,10)^ In our patient, stent placement was not selected primarily because the EBF had developed following palliative irradiation, and additional chemotherapy was planned as part of the ongoing treatment strategy. In addition, there was no evidence of tumor-induced esophageal obstruction, and the left main bronchial stenosis had been alleviated by the prior radiotherapy. The absence of significant luminal narrowing further supported the decision against stenting.

Bypass surgery is more invasive than stenting, and has been performed less frequently with the increased use of stenting, since the complication rate of bypass surgery is relatively high.^4,8,12)^ However, with recent advances in chemotherapy and radiotherapy, esophageal bypass surgery has been reevaluated as a viable option, as it provides superior long-term QOL compared with stenting.^8)^ Generally, the indications for esophageal bypass surgery include the following: (1) an ECOG-PS of 0–2; (2) an expected survival of more than 3 months; (3) a strong patient desire to maintain oral intake; (4) the presence of a large fistula; (5) a short segment of stricture or only mild stenosis; and (6) a planned course of CRT, among other clinically relevant factors.^4,9)^ Conversely, esophageal bypass surgery may be less appropriate in patients with: (1) swallowing dysfunction, such as that resulting from recurrent laryngeal nerve palsy or a history of radical neck irradiation; (2) lesions involving the cervical esophagus and/or bulky lymph node metastases, in which safe cervical anastomosis cannot be achieved; or (3) high surgical risk due to factors such as extensive pneumonia or severe malnutrition.^4,9)^ In the present case, the clinical background was carefully assessed in relation to these criteria. Although the patient had developed aspiration pneumonia secondary to an EBF, the condition was manageable with fasting and antibiotic therapy, and the ECOG-PS remained stable. Furthermore, the patient expressed a strong desire to resume oral intake. There was no evidence of recurrent laryngeal nerve palsy, swallowing function was preserved, and neither the primary tumor nor the metastatic lymph nodes involved the cervical esophagus. Based on these considerations, esophageal bypass surgery was deemed an appropriate treatment option. Moreover, the preoperative biopsy sample was small, and additional tumor sampling was needed to obtain the definitive diagnosis of malignant lymphoma. Unexpectedly, preoperative FDG PET/CT revealed newly enlarged lymph nodes at the lesser curvature of the stomach (**Supplementary Fig. A–C**). This finding enabled us to perform a lymph node biopsy during creation of the gastric tube, leading to the definitive diagnosis of malignant lymphoma.

Several methods of esophageal bypass surgery have been reported; however, a common challenge among these methods is the drainage of esophageal mucus and/or tumor secretions from the esophagus, which has become a closed space.^4,8,13,14)^ To manage secretions from the residual esophagus in our patient, a drainage tube was inserted via the distal esophageal stump to create an external fistula. Since only a minimal amount of drainage was observed, the tube was removed 6 weeks postoperatively. Several reports have suggested that in esophageal bypass surgery for patients with an EBF, simple closures of the oral and anal esophageal stumps have rarely led to the development of abscesses or mucoceles formation, as the remaining esophageal secretions drain through the fistula into the trachea.^3)^ In fact, follow-up CT in our patient confirmed the absence of an abscess in the thoracic esophagus even 4 months after bypass surgery (**Fig. 3B**–**3D**). Morishima et al. reported that closure of the anal side of the esophagus during esophageal bypass surgery was beneficial, as it helped prevent aspiration pneumonia caused by an EBF associated with vomiting.^3)^ It is important to note that the patient will require CT or FDG-PET monitoring, as endoscopic follow-up of the remaining esophagus is no longer possible after bypass surgery.

ERFs caused by malignancy are generally associated with a poor prognosis, with reported 6-month survival rates of less than 50%.^15)^ However, Nakajima et al. reported 1-year and 3-year overall survival rates of 45.7% and 15.3%, respectively, in patients who underwent esophageal bypass surgery for ERFs caused by esophageal cancer.^4)^ Moreover, patients with lymphoma-related ERFs tend to have a more favorable prognosis compared to those with ERFs caused by esophageal cancer, provided that appropriate treatment is administered.^3,16,17)^ In the present case, the EBF was caused by malignant lymphoma, and the patient fulfilled the previously described criteria for surgical intervention. As a result, the procedure improved our patient’s postoperative QOL and enabled subsequent chemotherapy. Esophageal bypass surgery should be actively considered for patients with EBF caused by malignant lymphoma, provided their condition allows it.

## CONCLUSIONS

We successfully performed esophageal bypass surgery for an EBF that became apparent after palliative radiation therapy for obstruction of the left main bronchus caused by a mediastinal malignant lymphoma. Esophageal bypass surgery is a valuable option for patients with malignant lymphoma and EBFs, as it improves their QOL and enables chemotherapy.

## SUPPLEMENTARY MATERIALS

Supplementary Fig (**A**–**C**) Preoperative FDG-PET/CT showed a marked reduction of the primary tumor and another new lesion on the lesser curvature of the stomach (yellow dotted circle).
