# Supplementary figures and images for: Esophageal Bypass Surgery for an Esophagobronchial Fistula Following Palliative Irradiation for Left Main Bronchial Obstruction Caused by Mediastinal Malignant Lymphoma
Source: Surg Case Rep. 2025 Sep 17;11(1):25-0246. doi: 10.70352/scrj.cr.25-0246 (PMC12450555; doi:10.70352/scrj.cr.25-0246)

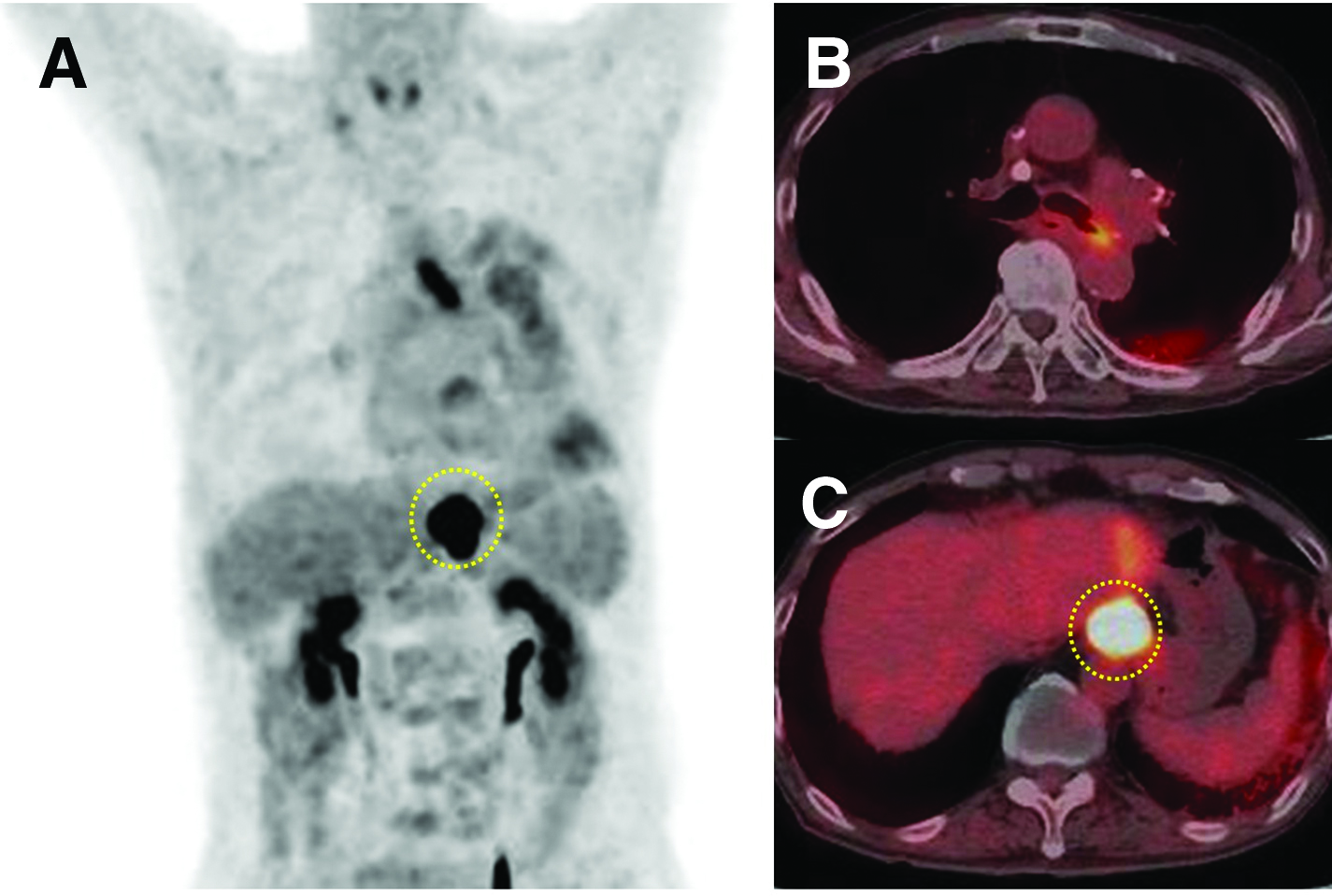

Supplement: Supplementary Fig [file scr-11-01-25-0246-s001.jpg]
